# Cyber-Enabled Optimization of HVAC System Control in Open Space of Office Building

**DOI:** 10.3390/s23104857

**Published:** 2023-05-18

**Authors:** Bo Peng, Sheng-Jen Hsieh

**Affiliations:** 1 Mechanical Engineering, Texas A&M University, College Station, TX 77840, USA; bopeng@tamu.edu; 2 Engineering Technology & Industrial Distribution (Joint Appt with Mechanical Engineering), Texas A&M University, College Station, TX 77840, USA

**Keywords:** cyber-physical system, HVAC, thermal comfort model, artificial neural network, support vector machine

## Abstract

Thermal comfort is crucial to well-being and work productivity. Human thermal comfort is mainly controlled by HVAC (heating, ventilation, air conditioning) systems in buildings. However, the control metrics and measurements of thermal comfort in HVAC systems are often oversimplified using limited parameters and fail to accurately control thermal comfort in indoor climates. Traditional comfort models also lack the ability to adapt to individual demands and sensations. This research developed a data-driven thermal comfort model to improve the overall thermal comfort of occupants in office buildings. An architecture based on cyber-physical system (CPS) is used to achieve these goals. A building simulation model is built to simulate multiple occupants’ behaviors in an open-space office building. Results suggest that a hybrid model can accurately predict occupants’ thermal comfort level with reasonable computing time. In addition, this model can improve occupants’ thermal comfort by 43.41% to 69.93%, while energy consumption remains the same or is slightly reduced (1.01% to 3.63%). This strategy can potentially be implemented in real-world building automation systems with appropriate sensor placement in modern buildings.

## 1. Introduction

Thermal comfort has been linked with personal happiness as well as worker productivity. Many people spend most of their time indoors. Indoor climate is almost exclusively controlled by heating, ventilation, and air conditioning (HVAC) systems. The energy cost of HVAC systems can account for up to 40% of the total energy usage in large cities [1]. Therefore, the design of HVAC systems in buildings is one of the most important aspects in building energy analysis.

EnergyPlus [2] and TRNSYS [3] are popular computing simulation software applications used to analyze complex building HVAC systems. These applications are preferred by engineers and scientists due to their superior calculation speed, realistic modeling capacity, and the convenience of rapid model development. However, even with these advanced features, implementing advanced control algorithms is often difficult. For example, control systems simulated in these applications are often limited to basic rule-based control, which is not sophisticated enough to fully capture the complexities of building systems. Additionally, occupant thermal comfort satisfaction is typically oversimplified to temperature regulation, which is not entirely accurate.

Thermal comfort is a sensation of thermal stress, mainly caused by human’s thermoregulatory effort of maintaining relatively constant body temperature. The modeling of thermal comfort typically can be categorized into three types: heat balance models, adaptive models, and data-driven models.

The most well-known heat balance model is the predicted mean vote (PMV) index, which is the current ASHERE and ISO standard [4]. The PMV index is a static model that uses six independent parameters (air temperature, mean radiant temperature, relative humidity, air speed, metabolic rate, and clothing insulation) to estimate human thermal comfort based on the heat exchange between human body and the surrounding environment, with heat dissipation from metabolism kept in mind. When the heat transfer rate reaches a certain level, the average thermal sensation (based on a large-scale field study) can be computed. The result is a seven-point scale representing user satisfaction level, which can range from −3 to +3 (too cold to too hot), where 0 is neutral.

Static heat balance models have been utilized in many thermal comfort research studies. For instance, Boregowda and Tiwari developed a human thermal model using finite element methods to simulate human thermal physiological responses and perception of thermal comfort in a non-uniform thermal environment [5]. Al-Mutawa, Chakroun, and Hosni conducted a large-scale experiment in Kuwait governmental offices to validate the existing ASHRAE PMV model proposed for use worldwide [6]. Results revealed that people in Kuwait felt comfortable with PMV values in the range of −0.5 to 0, which does not entirely correspond to the ASHRAE comfort zone. Additionally, people in Kuwait preferred an average operative temperature of 23 °C and relative humidity of 40%, while factors such as gender, age, and educational background appeared to have a negligible impact on thermal comfort preference. Farooq and Brown conducted a subjective survey to assess the thermal comfort level of students in an auditorium-type university classroom [7]. The surveys were analyzed using a regression method to predict the neutral temperature, preferred temperature, and acceptable temperature range for the students. This study also enables a comparison between thermal comfort level and energy consumption. Katramiz, Ghaddar, and Ghali investigated system performance for an office space application concerning thermal comfort and energy savings through the implementation of an appropriate control strategy. The results showed that the use of intermittent personalized ventilation (IPV) and mixed-mode ventilation (MMV) systems significantly reduced the number of AC operation hours while providing thermal comfort [8].

Adaptive models consider the preferences of different demographic groups. Previous research on adaptive models has examined factors such as age [9,10], gender [10,11,12], and fitness level [13]. Other factors, such as GDP per capita and floor area per person, have also been shown to influence people’s thermal sensations [14]. Typically, extensive survey or field studies are required to obtain representative results for these factors.

Recent advancements in ubiquitous sensor networks and machine learning provide an alternative approach to thermal comfort modeling: data-driven models [15]. In this approach, there is no predefined model, but rather models are generated based on inputs collected by a sensory system and feedback from users, who are the ultimate judges of thermal comfort. This approach has several benefits: (1) it can adapt to groups as small as one individual; (2) it is easy to incorporate into control systems; and (3) it reduces the need for highly accurate building modeling, which can be time-consuming and impractical for retrofitting HVAC systems.

Due to the vast number of variables and nonlinearity involved, machine-learning-based algorithms are preferred for modeling. Fuzzy logic [16] and artificial neural networks (ANNs) [17] have been explored for thermal comfort control, while deep learning methods such as convolutional networks [18] and long short-term memory (LSTM) networks [19] have been used for air quality prediction. Another supervised learning method, support vector machine (SVM), has also gained popularity. SVM has been applied in various fields, including building energy management [20], HVAC system diagnosis [21], and thermal comfort prediction [22]. Zhou et al. demonstrated that the SVM model could better fit data sampled from the ASHRAE RP-884 thermal comfort database than the PMV model [23].

The objective of this study is to enhance the performance of HVAC control by utilizing a data-driven thermal comfort model that is adaptable to occupant variability. This study is aimed at achieving the following research objectives:

- Develop a co-simulation platform based on a cyber-physical system (CPS) that can simulate building physics through specialized building simulation software while externally implementing advanced control algorithms.
- Establish a quantitative method of generating simulated building occupants using heat balance models and adaptive factors and determine the impact of occupant variability on HVAC control outcomes.
- Create a data-driven thermal comfort model and evaluate the accuracy of various machine-learning algorithms.
- Optimize the overall thermal comfort of multiple occupants in open-space office buildings using the proposed thermal comfort prediction model and assess its effectiveness.

## 2. Materials and Methods

### 2.1. Overview of Methods

#### 2.1.1. Co-Simulation

To overcome the limitations of traditional building simulation programs, in this research, a co-simulation of EnergyPlus and MATLAB are coupled within the middleware Building Controls Virtual Test Bed (BCVTB) developed by Lawrence Berkeley National Laboratory [24] and MLE+ developed by University of Pennsylvania [25].

**Figure 1:**
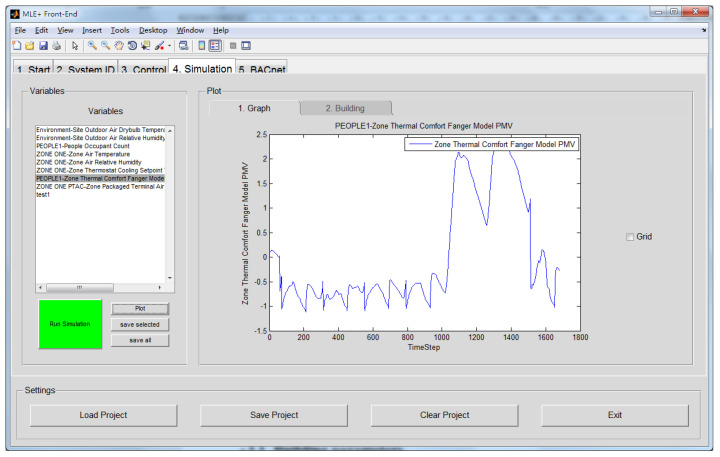
Coupling of MATLAB and EnergyPlus in MLE+.

EnergyPlus is used to simulate the realistic behavior of buildings, and MATLAB is used to control the actuators (air conditioners) in the rooms based on the optimization of the data generated by EnergyPlus. BCVTB and MLE+, as middleware, provide a user-friendly interface to integrate these two different tools (Figure 1).

#### 2.1.2. Control System

**Figure 2:**
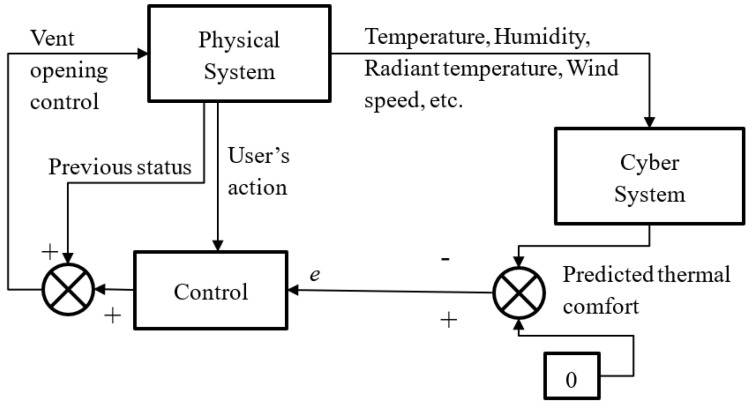
Control diagram for system.

The objective of the control system is to minimize thermal comfort unsatisfaction and potentially the energy cost of the HVAC. The control system used in this research is a feedback closed-loop control system. The diagram is shown in Figure 2.

#### 2.1.3. Control Strategies

The control strategy is defined as a combination of the thermal comfort model and control algorithms. The former defines the criteria for the thermal comfort of the occupants, and the latter decides how the predicted comfort level is used to control the physical system. Three control strategies are defined and used in this research.

Conventional rule-based temperature control. In conventional rule-based control, the thermal comfort objective is based purely on temperature to fulfill ventilation requirements, with potential limitations due to humidity. The actuators in the HVAC system (vents) are then controlled according to the set point.

Thermal comfort-based control with probability model. In the thermal comfort-based control with probability model strategy, thermal comfort level is evaluated based on the air temperature and relative humidity of the room using probabilistic reasoning. In implementation, the indoor temperature at which the user is comfortable (for example, when there is no control action for a period) is recorded in the first phase. When enough data are collected, a probability distribution is used to predict the most comfortable setting. Mathematically, this strategy is essentially a regression model. This method is used in some commercial thermostat products.

Thermal comfort-based control with machine-learning model. The strategy of using a machine learning model for thermal comfort-based control is somewhat similar to the probability-based strategy. In the training phase, multiple environmental parameters, including air temperature, humidity, radiant temperature, and user input, are collected and used to train a personalized model for a specific occupant’s comfort. Unlike a simple probability model that considers only temperature and humidity as parameters, this model is derived from a standard physical heat-balanced model of the human body, known as the PMV index, and includes consideration of adaptive factors such as gender, age, and BMI. Once the model is trained, the predicted thermal comfort level serves as the control variable for HVAC control. A fuzzy control set is used as the control core for the controller, and thermal comfort is divided into five levels: cold, neutral, warm, hot, and extremely hot. Since the output variable is the opening of the vent, which is a continuous value, the output is evaluated and applied to a PID control.

These three strategies are summarized in Table 1.

### 2.2. Experiment Setups

#### 2.2.1. Building Simulation

The building model is based on a real room, with a view toward calibration in later steps.

The size of the room is 6.89 m × 6.83 m × 2.44 m (or 22.6 ft × 22.4 ft × 8 ft, L × W × H). It has one large 8 m × 3 m window facing north. The HVAC system is a typical central HVAC VAV box system. The air temperature varies from 16 to 22 °C. The heat dissipation of occupants dynamically changes based on the sum of metabolic rates obtained from EnergyPlus ActLevel variable in the occupant simulation. Other heat sources included 1500 W lights and 1200 W electric equipment (computers, etc.) The properties of the building materials are listed in Table 2.

Six cubicles were formed, each 0.61 m × 0.61 m (2 ft × 2 ft), in the middle of the room, to mimic the layout of a typical office. Two types of boundaries were used to separate each zone: First, the boundaries between Zones 1 to 6 and the outer space Zone 7 constitute “air gaps”, to simulate the dynamics of each cube in a large open space while maintaining behavior comparable to a single continuous space. Second, two boundaries between each cube are semi-walls, which will be calibrated later.

**Figure 3:**
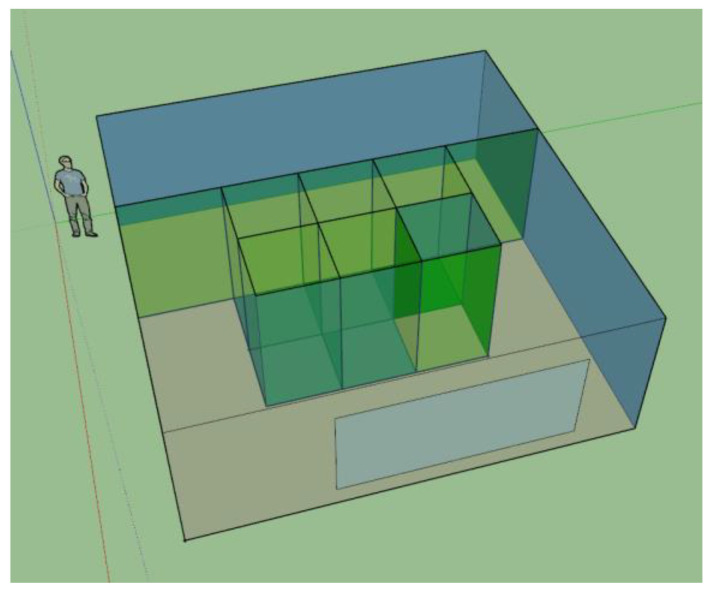
The 3D model built in SketchUp with virtual walls showing.

**Figure A1:**
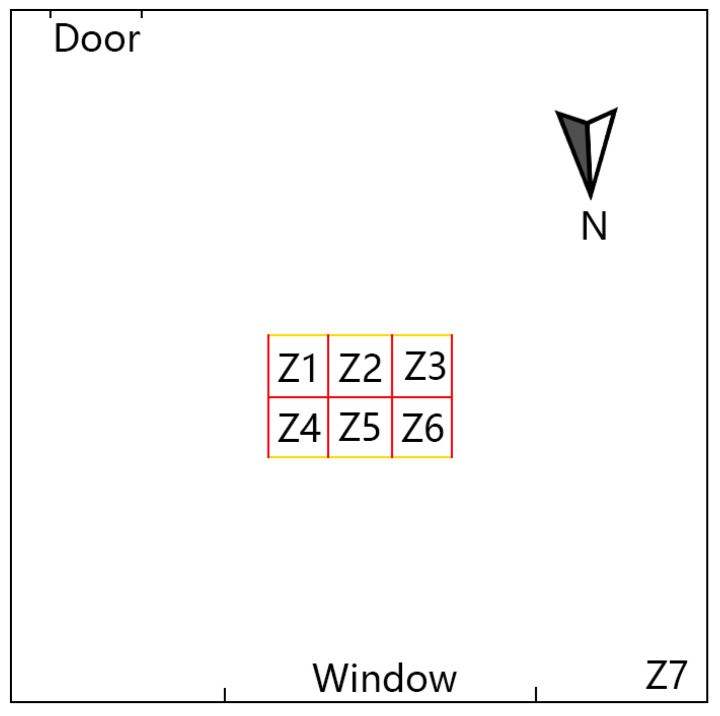
Floor plan of the simulated building room.

The simulation model was drawn using SketchUp with the above parameters (Figure 3) and imported into EnergyPlus. Figure A1 in Appendix A shows the floor plan of the simulated room.

For weather conditions, historical weather data from dataset Typical Meteorological Year 2/3 provided by National Renewable Energy Laboratory [26] for four U.S. cities were used (Table 3). To evaluate the flexibility of the system, cities from different regions were selected. In addition, because the focus of this study is on air conditioning, most of the cities chosen are located in the southern U.S.

The influence of exterior solar radiation, especially through the window in the building, is simulated using EnergyPlus built-in solar options, based on Horizontal Infrared Radiation Intensity provided in the weather file. The ASHRAE Clear Sky solar model is used.

#### 2.2.2. Occupant Simulation

The adaptive models described below are used to simulate profiles or models of occupants. The primary objective is to generate occupants that are realistic and varied enough to test the machine-learning model’s flexibility. As such, only a few typical adaptive factors are considered instead of an exhaustive list of variables.

The mathematical model of each occupant is represented by the deviation of their thermal comfort from the standard heat balance model (PMV index model is used in this study), due to adaptive factors, in two ways: (1) shift their comfortable region (i.e., at what kind of thermal environment they feel comfortable) and/or (2) change the range of the region (i.e., how far away from the neutral point they will start to feel uncomfortable).

The first part can be represented by a neutral modified temperature, which means at this specific *T_m_*, subject feels most comfortable:

$$T_{m,neu}=T_{m,neu,region}+\Delta T_{m,neu,gender}+\Delta T_{m,neu,fitnss}$$

where modified temperature *T_m_* is the equivalent temperature to factor in all the environmental factors into one based on predicted mean vote (PMV) model.

The base $T_{m,neu,region}$ is decided by the region that the occupants come from. In this study, it is assumed from the US [27], which is 25.6°.

The female feels colder within the same temperature than the male. In addition to different mean neutral points, the female tends to have slightly wider variance in terms of their preference [10,11,12,13]. Table 4 lists the equations adopted in this research.

Deviations from neutral temperature for different fitness level based on BMI (body mass index) [13] are listed in Table 5.

For comfortable region range, the relationship from [28] is used to calculate the comfortable range of young and elderly people:

Young: $Comfort\_region=\left[T_{neu}-\left(1.0\pm 0.4\right),T_{neu}+\left(0.8\pm 0.4\right)\right]$

Elderly: $Comfort\_region=\left[T_{neu}-\left(2.2\pm 1.2\right),T_{neu}+\left(1.9\pm 0.5\right)\right]$

**Figure 4:**
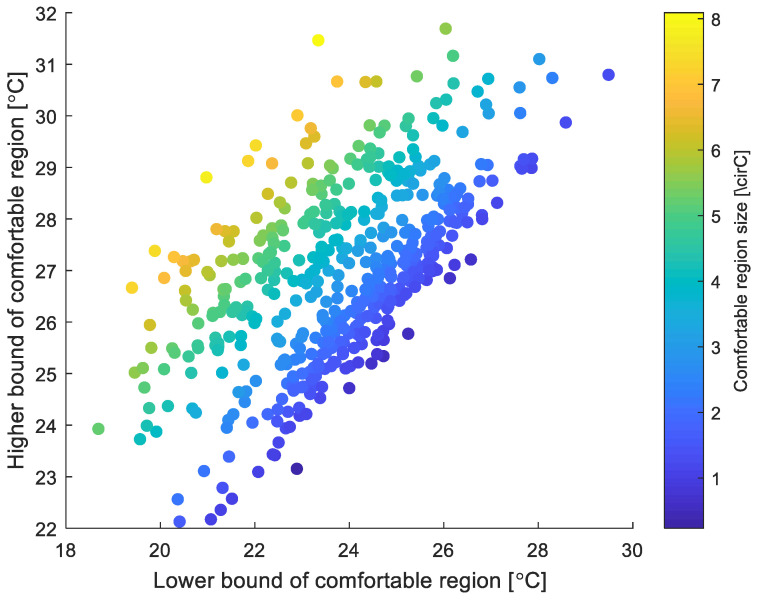
Comfortable regions’ lower and upper bounds for 500 generated subjects.

**Figure 5:**
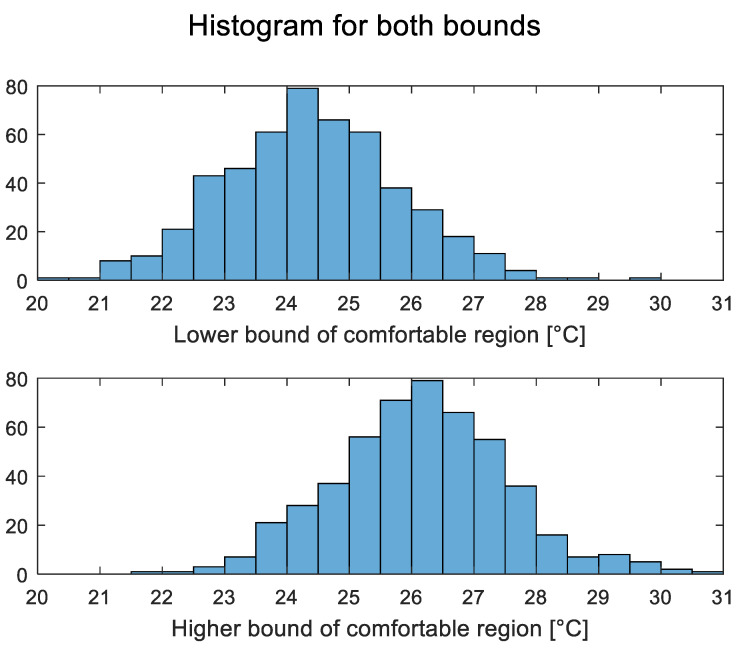
Histogram of comfortable regions’ (**lower**) and (**upper**) bounds of 500 generated subjects.

The minimum comfort region size is considered 0.5 °C modified temperature. Any generated data that are less than this will be expanded in both directions until the minimum is reached. Figure 4 and Figure 5 below show data for 500 subjects randomly generated using the above rules.

Once the occupants are generated, a total of six occupants are selected. The comfort level can be interpolated from the comfortable region above using the three fixed points: *T_m,neu_* and the lower and upper bounds, with current modified temperature, *T_m_*.

*T_m_* is calculated using environmental inputs. In short, *T_m_* is the temperature that will give the same PMV value when all the other heat balanced factors are set as standard value. It can be calculated by recursively solving the following equation:

$$PMV\left(T_{m},T_{m},RH_{std},f_{cl,std},W_{std},M_{std},V_{a,std}\right)=PMV\left(T_{a},T_{mrt},{RH,f}_{cl},W,M,V_{a}\right)$$

On the left, both air temperature and mean radiant temperature use modified temperature *T_m_* (i.e., assuming they are the same). The other parameters are fixed as standard values: relative humidity *RH_std_* = 0.4; clothing factor *f_cl,std_* = 0.6 clo; external mechanical work *W_std_* = 0 W/m^2^; metabolic rate *M_std_* = 1 Met; mean radiant temperature *T_mrt_* = Tair; and air velocity *V_a,std_* = 0.1 m/s.

The environmental inputs used are listed in Table 6.

### 2.3. Procedures

**Figure 6:**
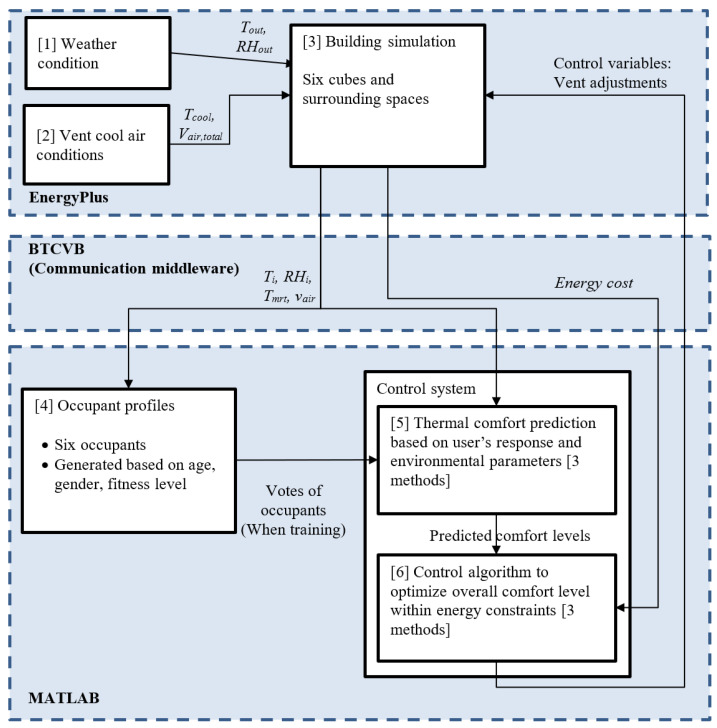
Overall system structure and workflow.

The overall simulation procedure is described below and depicted as a flowchart in Figure 6.

1. Randomly generate six subjects to use, following the rules above. The features (gender, age, and fitness level) are uniformly chosen.
2. Assign each subject to a cubicle in the simulated building.
3. The building physical model is simulated in EnergyPlus, with the environmental data pipelined to MATLAB.
4. The simulation period spans 6 months, from 1 May to 31 October. The corresponding weather data are provided by the EnergyPlus database, which is the average historical data for each location.
5. For the probability model and machine learning model, the first few days are used as training phases. The thermal comfort level of each subject represents their “votes.”
6. After the model is trained, the predicted level from the thermal comfort model is used as an input to the control algorithm. The values of the control variables at the conclusion of each cycle are pipelined back to EnergyPlus as settings for the actuators (set point, volume flow rate of each vent in each cube) for the next cycle.
7. For the machine-learning-based modeling methods, the trial is repeated 500 times for each city with different random seeds.

## 3. Results

### 3.1. Experimental Calibration of the Simulation Model

The thermal properties of the partition walls between cubes were calibrated using experiments (Table 7).

**Figure 7:**
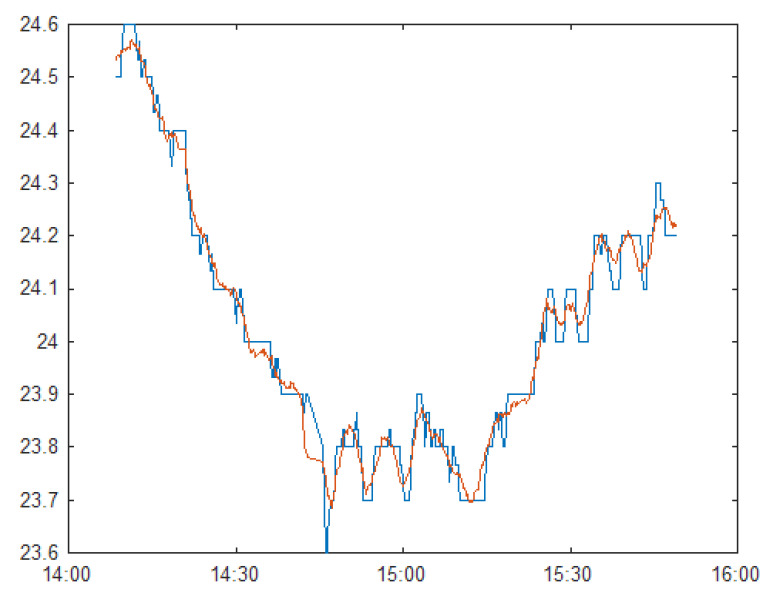
Comparison of measured temperature (blue) with simulated temperature (red), with 0.043 m^2^-K/W as virtual wall resistance.

A physical setup identical to the simulation model was built in an office space, with temperature and humidity sensors placed in each cubicle to record data. The temperature and humidity readings from one zone (5) were fixed in the simulation, and readings from the other zones were compared with experimental results (Figure 7). The resistance of the partition wall is repeatedly adjusted to achieve the best fit. To prevent overfitting, only data from Zone 4 and 6 are used in the calibration process.

After the calibration experiment, the virtual wall resistance was determined to be 0.043 m^2^⋅K/W (indicated in bold in Table 7)

### 3.2. Thermal Comfort Level

Four different regressors were tested for prediction of continuous comfort level: least squares (LS), binary decision tree (BDT), artificial neural network (ANN), and support vector machine (SVM). The parameter settings for each method are described in Table 8. The training dataset size varied from 50 to 200. The upper limit of dataset size is selected based on preliminary results to ensure it achieves sufficient prediction accuracy without overfitting. Two lower values are selected to examine the quality of the regression when samples are few, since shorter training data collection time is desired in practice. Each model was hyperparameter-optimized using a small trial set of data. L2 regularization was also applied to the least squares method.

#### 3.2.1. Training and Validation

Metrics used included training computing time, resubstitution mean squared error (MSE), and resubstitution coefficient of determination (*R*^2^) for training; these were evaluated for all regressors.

The SVM regression model outperformed all the other models in terms of accuracy (Table 9). It had significantly lower MSE when using small (50 or 100) data sets. The ANN model reached similar levels of performance when the size of the data set increased to 200. Both the SVM and ANN models can reach around 0.99 R^2^ with only 50 data points. Statistically, the difference between the two is not significant.

**Figure 8:**
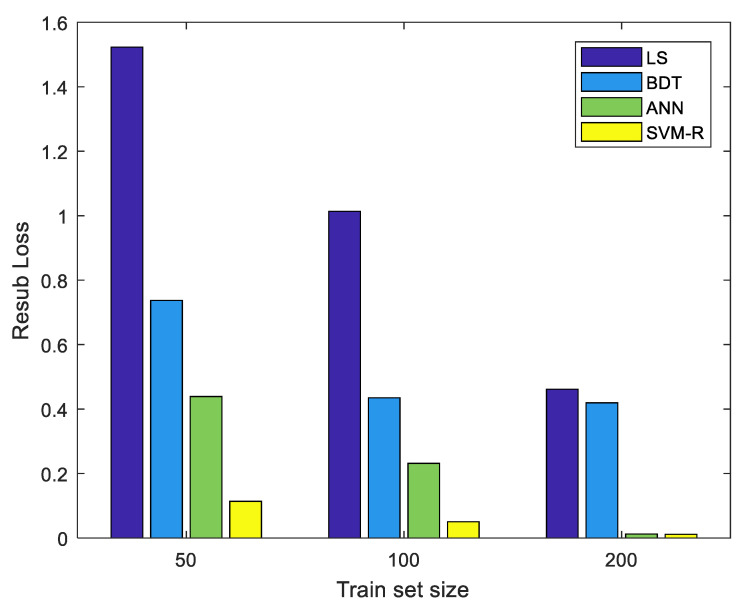
Resubstitution MSE of various training data set sizes for least squares, binary decision tree (BDT), artificial neural network (ANN), and support vector machine (SVM).

**Figure 9:**
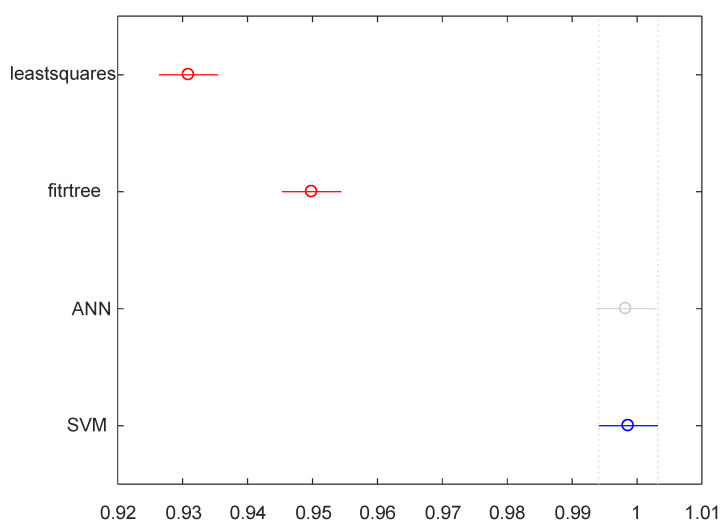
One-way ANOVA test between the groups on R^2^ (N = 200). Group Least Squares and Binary Decision Tree (red) have means significantly different from group SVM (blue).

The performance of the BDT model is relatively poor compared to the ANN and SVM and does not improve much when the size of the dataset is increased (Figure 8 and Figure 9).

**Figure 10:**
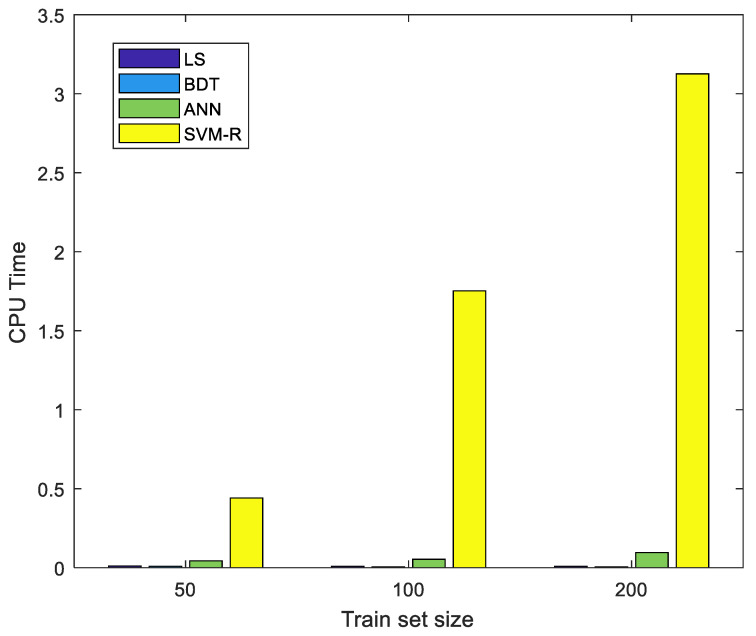
Computing time for various training data set sizes for least squares (LS), binary decision tree (BDT), artificial neural network (ANN), and support vector machine (SVM).

Figure 10 shows that SVM’s complexity hurts its efficiency, as its computing time is hundreds of times longer than that of the other algorithms. When dataset size increased to 200, the SVM model required 438 times more computing time than the least squares algorithm and 39 times more than artificial neural network.

On the other hand, the ANN algorithm shows great potential. Its accuracy is on par with that of the SVM when the training dataset size is 200, while the computing time is only a fraction of that of the SVM. This is due to the different underlying principles of the two algorithms: the backpropagation ANN is more suitable for regression problems; the SVM regression is less efficient, as it is based on classification.

#### 3.2.2. Prediction Test Performance

After the training, 3000 more data points were used to test the performance of the models. Mean squared error (MSE) and coefficient of determination (*R^2^*) again were used to evaluate the results.

Results are listed in Table 10. One obvious difference is that the BDT algorithm had a substantially worse outcome. This suggests that the previous training model had an overfitting issue. The other algorithms performed similarly in both the test and training sessions. The ANN had better MSE and *R^2^* with 200 training data points than the SVM, but the difference is small.

#### 3.2.3. Hybrid Thermal Comfort Model

**Figure 11:**
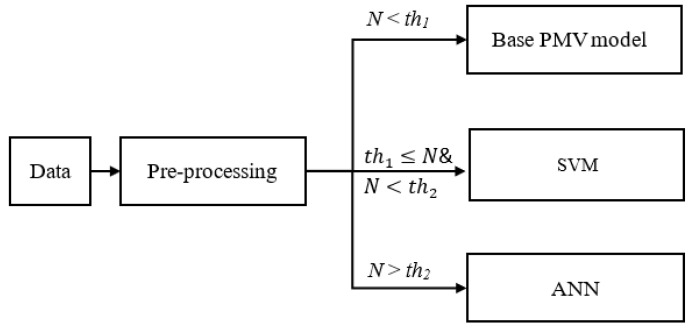
Flowchart of hybrid model (*N*: training set size; *th_1_*: threshold 1, *th_2_*: threshold 2).

The efficiency difference between the SVM and ANN algorithms inspired us to build a hybrid model that can leverage both the rapid computing time of the ANN and the high accuracy of the SVM when using small training sets (Figure 11).

Additionally, data-driven models are limited in that they are not operational until training is complete. In this research, a predicted mean vote (PMV) model is used as a placeholder for the thermal comfort model until enough data points are acquired.

Table 11 shows the average thermal comfort across three different periods within the beginning of the simulation. As expected, the hybrid shows good results in all periods, giving the best or very close to the best result.

#### 3.2.4. The Influence of Noise and Delay in Data Sensing

To realistically simulate the behaviors of sensors, including low fidelity, time delay and inaccuracy caused by poor placement, a layer of noise was added to the measured environmental data from EnergyPlus.

**Figure 12:**
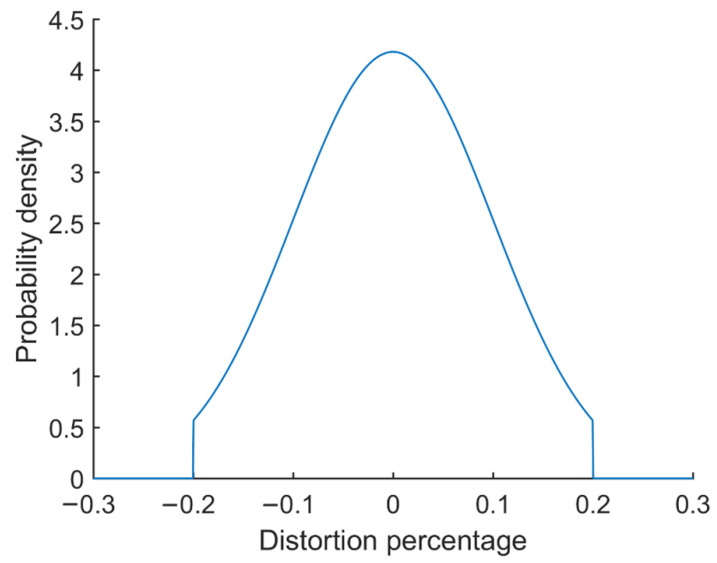
Probability density function of noise distribution when X = 20%.

The noise consists of two parts:

1. Distortion up to a certain percentage X is added to the temperature and humidity values before they are fed into the thermal comfort prediction model. The probability model of the distortion is a two-sided truncated normal distribution with mean equal to 0 and sigma set to X/2 (Figure 12).
2. A time delay between the moment the sensing data is measured and the moment the data is used to build the thermal comfort model.

The test was repeated for noise levels *X* = 5% to 20% and time delays of reading = 6 or 12 min, applied to both temperature and humidity readings.

The result is summarized in Table 12. The significance is based on an independent two-sample *T*-test on the mean of MSE, where the control group is without noise.

A small percentage of error (less than 5%) in environment readings is acceptable and will not change the accuracy of thermal comfort prediction significantly until it exceeds 10%. For temperature, 5% typically means around 1 °C. Most sensors today, even lower quality ones, can easily reach <0.2 °C accuracy, so this is not a significant concern for applications.

On the other hand, a time delay in reading could have substantial effect on the modeling. Even a one cycle delay (6 min) causes significant decline in accuracy of the modeling. One interesting observation is that the effect of time delay is relatively constant rather than being proportional to the length of the delay (Table 13). This is likely because changes in temperature and humidity in a room are relatively slow and stable.

The last two columns show the equivalent error introduced by the time delay in two readings, which follow a similar trend.

### 3.3. Control Performance

After the thermal comfort model was finalized, the main simulation was conducted with the variables shown in Table 14.

Three thermal comfort models were used: a model based on conventional temperature and humidity, an SVM (the most performant algorithm in thermal comfort model evaluation), and the proposed hybrid model.

For control strategy, rule-based control was achieved by using the built-in thermostat feature in EnergyPlus. The fuzzy-PID control is the proposed control strategy utilizing predicted thermal comfort level in an external MATLAB program. Each batch of the simulation ran for 500 trials, except for T- and RH-based control, which is deterministic.

#### 3.3.1. Overall Thermal Comfort Level and Energy Cost

**Figure A2:**
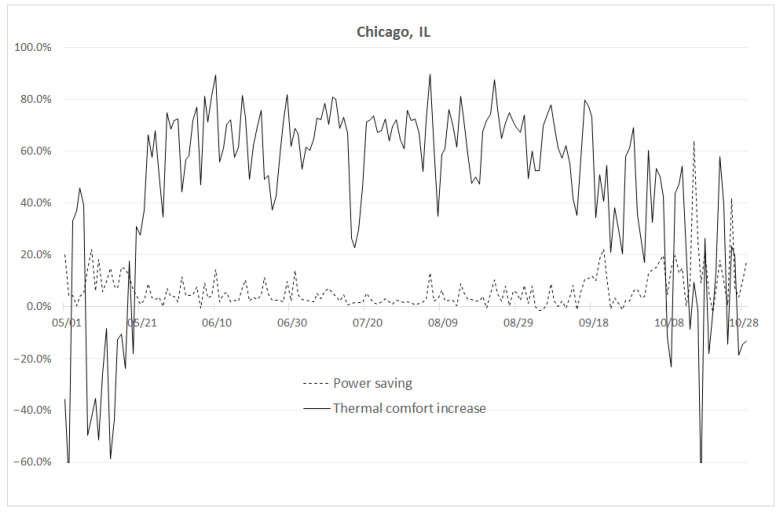
Thermal comfort increase and energy savings in Chicago, IL.

**Figure A3:**
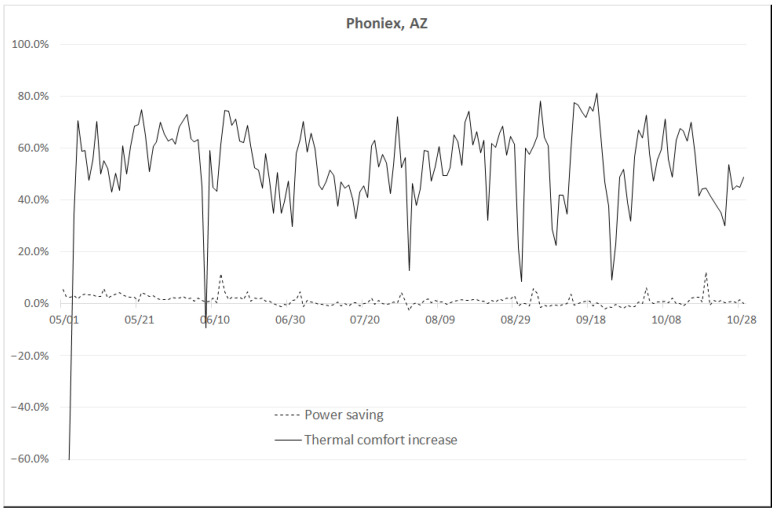
Thermal comfort increase and energy savings in Phoenix, AZ.

**Figure A4:**
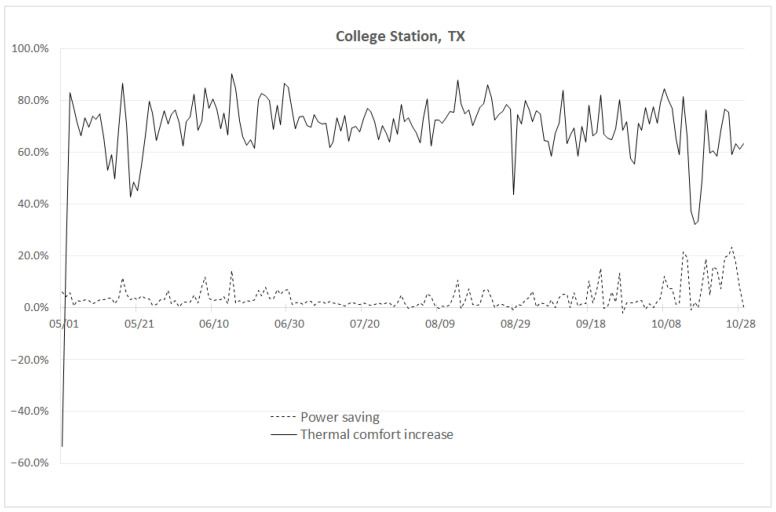
Thermal comfort increase and energy savings in College Station, TX.

**Figure A5:**
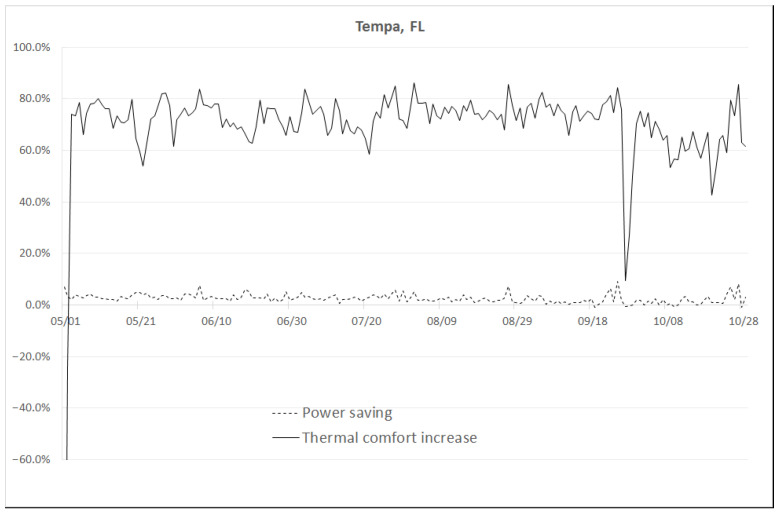
Thermal comfort increase and energy savings in Tampa, FL.

The proposed system was compared with a baseline (conventional rule-based control based on temperature control). Figure A2, Figure A3, Figure A4 and Figure A5 in Appendix A show the trend of energy saving percentage and mean absolute thermal comfort level (the lower, the better) reduction percentage for four US cities (Chicago, IL; Phoenix, AZ; College Station, TX; and Tampa, FL). The data show significant thermal comfort improvement in all four cities. The energy cost remains the same or is slightly improved.

The monthly data are summarized in Table 15. On average, 43.41% to 69.93% reduction in thermal comfort level (i.e., increase in comfort) was achieved. The energy cost change is small by comparison, ranging from 3.63% to 1.01%.

In the hybrid model, the thermal discomfort increases at the beginning of the model-building process but is limited to the first one to three days. The length of the poor performance period is noticeably longer in Chicago, IL, which is likely due to the relatively cool climate. If the air conditioner is not actively engaged, it is difficult to influence the thermal comfort level of the occupants. Cooler climate also has a detrimental effect on model training: a more moderate outdoor climate means that the difference between indoor and outdoor is not as dramatic as in hotter climates. Without a wide variety of input and output ranges, the training process is prolonged.

**Figure 13:**
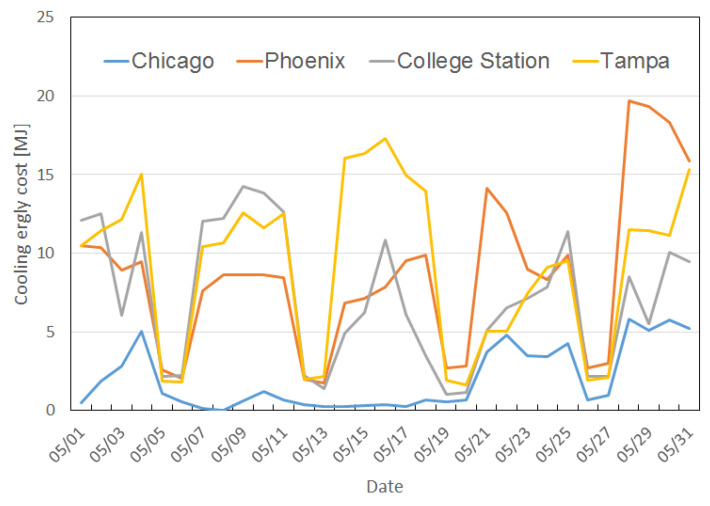
Cooling energy cost in May in four cities.

For reasons described above, the overall result in Chicago fluctuates more in comparison with the other cities, and the improvement is relatively low in May and October due to less demand for air conditioning. Figure 13 highlights the difference in cooling demand between the four cities.

#### 3.3.2. Effect of Thermal Comfort Models

To test how the thermal model affects control performance, the proposed hybrid model was compared with the SVM-R only prediction model, together with the probability model based on temperature and humidity. All these models were paired with the same fuzzy-PID control system. Baseline rule-based control data are also listed for comparison purposes. Both baseline and the probability cases have no random number involved in simulation, so the data are presented as is. The models based on machine learning (ML) were run 500 times with mean and standard deviation listed.

Table 16 reveals that all three models (basic probability-based model, ML model with the SVM-R algorithm, and ML model with the proposed hybrid model) showed significant improvement in thermal comfort over the baseline conventional strategy. The probability-based model, which utilized temperature and humidity alone, brought the thermal comfort level from 0.2217 to 0.1451. The machine-learning-based model reduced thermal comfort level to 0.1003 with the SVM-R and 0.0942 with the hybrid model.

In addition, the cooling energy costs (last row in Table 16) remained relatively stable. This is likely because the capacity of the HVAC system is constant (the sizing of compressor, cool air temperature, and total air flow rate remain the same); the new control strategy simply allows better cooling distribution between the cubes, rather than an overall reduction of cooling effort.

**Figure 14:**
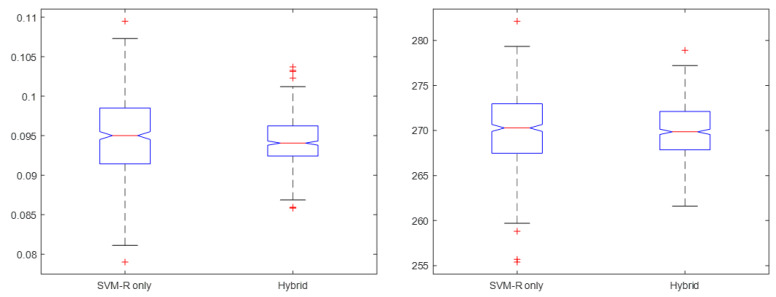
Box plots of the data for SVM-R only and hybrid control strategy (**left**: comfort level, **right**: energy cost).

In statistical analysis, an ANOVA test of the means of the two tested ML models shows that the lowering of (dis-)comfort level is significant between the two treatments (*p* < 0.05), but not between the energy costs (Figure 14 and Table 17). It should be noted that improvement is present, but the difference is small.

#### 3.3.3. Variances in Zones and Subjects

The parameters of the six subjects generated in the main experiment batch are listed in Table 18 below.

Table 19 shows the average behavior of the six occupants in the College Station, TX batch with the proposed system. The manual action time means the simulation step where the ground truth of the occupants’ thermal comfort is far enough away from the neutral point to cause them to adjust the thermostat.

In general, the trend of the manual action time is consistent with average thermal comfort level: the lower the comfort level, the less likely it is that a particular occupant is going to adjust their settings.

The overall reduction in thermal discomfort before and after training is relatively stable, except for subject No. 5, who shows much greater improvement than the others. This can be attributed to her “comfort region” being relatively wider.

The average comfort level for subject No. 6 after training is 0.161, which is much higher than the average 0.094. This is caused by the fact that her neutral modified temperature is much higher than others. Because the cubes are close together, and the air exchange between them is high, optimizing for the average of the six objectives results in her staying in a relatively “cool” local climate. This is the only limitation of the system in this research.

While not actively maintained or monitored, as an observation, the humidity of the six zones is relatively uniform in the simulation. Mostly they stay within a 5% RH window around 38%. Because the cubes ventilate cool air directly into the space, the temperature drops relatively quickly when the vent is opened.

## 4. Conclusions

In this study, a novel data-driven thermal comfort model and HVAC control system were implemented in an open-space office room. An adaptive occupant simulation model was utilized to evaluate the results.

The evaluation of the thermal comfort prediction model revealed that the SVM and ANN algorithms were competitive, achieving a convergence of 0.99 *R^2^* in both training and testing. However, the SVM required a longer training time, making it less suitable for low-power controllers. In addition, data-driven models performed poorly in cold-start scenarios, leading to the construction of a multi-stage hybrid model that transitioned from a PMV model to SVM and then to ANN as the number of data points increased. This model demonstrated promising results in cold-start scenarios.

The model’s prediction was then used as a control objective in a 6-month simulation of a full control system in four cities. The model reduced thermal discomfort by 43.41% to 69.73% across all cities and resulted in energy savings of 1.01% to 3.63%. Additionally, three thermal comfort models were compared, with the hybrid PMV-SVM-ANN model showing a statistically significant improvement over the SVM-only model. Both models outperformed the basic temperature-and-humidity-based probabilistic model. Finally, the variance between occupants was investigated, and the system’s limitations in open-space settings were discussed.

The system is relatively simple to set up, and the computing cost for running the system is minimal. The initial training can be performed using a computer. Afterwards, the model and control algorithms can be integrated into typical commercial building energy management systems using embedded microcontroller add-ons or smart thermostats via an API.

Future directions for this research include implementing more advanced control algorithms such as predictive control, as well as online learning in modeling, to further enhance the performance of the HVAC control system. The authors have already fitted the system into an office room to conduct experimental analysis of the proposed work’s effectiveness, and plan to expand it to large open-space buildings. A comparison with conventional CFD models in building simulation is also planned.

## Figures and Tables

**Table 1:** Control strategies.

Thermal Comfort Model	Inputs	Available Control Algorithms
Traditional	*T, RH*	On/Off
Probability-based	*T, RH*	On/Off, PID, Fuzzy
Machine-learning-based	*T, RH, T_mrt_, T_cool air_, v_air_*	On/Off, PID, Predictive

**Table 2:** Construction material properties in simulated building.

Construction	Material	Major Thermal Properties
Wall	R13 Layer	Thermal resistance: 2.291 m^2^⋅K/W
Roof	R31 Layer	Thermal resistance: 5.456 m^2^⋅K/W
Floor	HW Concrete	Conductivity: 1.729577 W/(m⋅K) Specific heat: 836.8 J/(kg⋅K)
Window	Glass (6 mm)	Conductivity: 0.9 W/(m⋅K) Transmittance: 0.81

**Table 3:** List of cities used in the simulation experiment and their locations.

City Name	Location	Elevation (m)	Data Sources
Phoenix, AZ	N33°25′, W112°1′	+339	WMO Station 722780
Chicago, IL	N41°58′, W 87°55′	+201	WMO Station 725300
College Station, TX	N30°34′, W 96°22′	+96	WMO Station 722445
Tampa, FL	N27°58′, W 82°31′	+6	WMO Station 722110

**Table 4:** Influence of gender on neutral modified temperature deviation.

Gender	$\Delta T_{m,neu,gender}$
Female	−0.5 ± 1.3
Male	0.4 ± 1.4

**Table 5:** Influence of fitness on neutral modified temperature deviation.

Fitness (BMI)	$\Delta T_{m,neu,fitness}$
Thin (<20)	0.3
Normal (20–24)	0
Fat (>24)	−0.5

**Table 6:** Environmental inputs for occupant thermal comfort model.

Parameter	Unit	Lower Limit	Upper Limit
Room temperature	°C	20	28
Mean radiant temperature	°C	20	28
Relative humidity	(n/a)	10%	90%
Air speed	m/s	0	0.3

**Table 7:** Results of calibration.

Wall Resistance (m^2^⋅K/W)	Correlation R^2^		
	Average	Zone 4	Zone 6
0.01	0.67	0.57	0.62
**0.043 (best)**	**0.87**	**0.86**	**0.90**
0.1	0.74	0.79	0.69

**Table 8:** Parameter settings for open-space regression methods.

Method	Abbreviation	Parameters
Linear (least squares)	LS	*L*_2_ regularization applied Regularization: ridge
Binary decision tree	BDT	Min leaf size: 2 Max number splits: 30
Artificial neural network	ANN	Backpropagation algorithm: Levenberg–Marquardt Hidden layer size: 10 Validation dataset ratio: 20%
Support vector machine regression	SVM	Kernel function: Gaussian Kernel scale: 6.68 Box constraint: Inf Epsilon: 0.008 Standardized Outlier fraction: 20%

**Table 9:** Training resubstitution coefficient of determination R^2^.

Train Set Size	Least Squares	Binary Decision Tree	Artificial Neural Network	Support Vector Machine
50	0.7947	0.9092	0.9715	0.9897
100	0.8924	0.9366	0.9913	0.9970
200	0.9309	0.9499	0.9983	0.9987

**Table 10:** Prediction test performance of regression models.

Training Dataset Size	Least Squares		Binary Decision Tree		ANN		SVM	
	MSE	R^2^	MSE	R^2^	MSE	R^2^	MSE	R^2^
50	1.7189	0.7700	4.1075	0.6330	0.8143	0.9117	0.4321	0.9643
100	1.1663	0.8852	1.7640	0.7233	0.4622	0.9773	0.2574	0.9815
200	0.4868	0.9267	1.3835	0.7896	0.0289	0.9962	0.0592	0.9935

**Table 11:** Average resub-MSE on cold-start in August in College Station, TX.

Period	Static PMV Model	SVM Only	ANN Only	Hybrid (Proposed)
First day	0.482	0.928	1.708	0.484
Second day	0.466	0.332	0.606	0.336
Day 3–5	0.462	0.200	0.366	0.202
First work week average	0.47	0.278	0.468	0.244

**Table 12:** Average accuracy drops in model training due to noise.

Maximum Noise Percentage	Accuracy Drop in R^2^	Accuracy Drop in MSE	Significant? (*p* < 0.01)
5%	1.22%	1.01%	No
10%	2.02%	2.49%	Yes
15%	4.88%	4.10%	Yes
20%	15.50%	19.00%	Yes

**Table 13:** Average accuracy drops due to time delay.

Time Delay (Min)	Reduction in Training R^2^	Reduction in Training MSE	Significant? (*p* < 0.05)	Accuracy Drop in Temperature Reading	Accuracy Drop in Humidity Reading
6	3.03%	3.65%	Yes	2.88%	3.14%
12	4.03%	3.80%	Yes	3.20%	3.56%
18	4.11%	3.99%	Yes	3.70%	3.88%

**Table 14:** Summary of simulation variables in main batch.

Batch	Thermal Comfort Model	Control Strategy	City	Trial (Total)	Note
1–4	(T and RH)	Rule-based	4 cities	4	Conventional
5–8	(T and RH)	Fuzzy-PID	4 cities	4	Probability-based
9–12	SVM-only	Fuzzy-PID	4 cities	2000	
13–16	Hybrid	Fuzzy-PID	4 cities	2000	Proposed

**Table 15:** Mean absolute thermal comfort level and energy cost in cooling in different models.

City	Month	Monthly Mean Absolute Thermal Comfort Level			Monthly Cooling Energy Cost (MJ)		
		Baseline	Proposed	Reduction	Baseline	Proposed	Reduction
Chicago, IL	May	0.235	0.203	13.84%	64.628	61.624	4.65%
	June	0.236	0.081	65.79%	155.881	149.508	4.09%
	July	0.194	0.066	65.74%	266.506	259.994	2.44%
	Aug.	0.230	0.073	68.40%	164.487	158.317	3.75%
	Sept.	0.301	0.140	53.70%	88.348	85.282	3.47%
	Oct.	0.460	0.375	18.46%	19.718	17.217	12.69%
	Ave/Sum	0.276	0.156	43.41%	760.568	732.942	3.63%
Phoenix, AZ	May	0.181	0.102	43.63%	276.985	269.030	2.87%
	June	0.158	0.069	56.12%	487.225	480.375	1.41%
	July	0.154	0.074	51.66%	525.389	523.556	0.35%
	Aug.	0.169	0.080	52.44%	484.505	481.204	0.68%
	Sept.	0.224	0.105	52.98%	315.150	314.920	0.07%
	Oct.	0.272	0.132	51.64%	173.904	171.283	1.51%
	Ave/Sum	0.193	0.094	51.37%	2264.158	2241.368	1.01%
College Station, TX	May	0.199	0.073	63.21%	232.391	224.570	3.37%
	June	0.221	0.050	77.56%	296.316	284.955	3.83%
	July	0.149	0.043	70.85%	425.060	416.939	1.91%
	Aug.	0.199	0.051	74.56%	394.577	387.364	1.83%
	Sept.	0.238	0.071	70.13%	197.880	190.579	3.69%
	Oct.	0.368	0.129	64.92%	136.967	129.460	5.48%
	Ave/Sum	0.229	0.069	69.65%	1684.191	1634.867	2.93%
Tampa, FL	May	0.180	0.069	61.56%	295.717	286.487	3.12%
	June	0.173	0.047	72.94%	328.932	318.950	3.03%
	July	0.173	0.046	73.62%	401.569	390.060	2.87%
	Aug.	0.177	0.043	75.86%	382.716	374.504	2.15%
	Sept.	0.200	0.057	71.57%	305.316	301.425	1.27%
	Oct.	0.230	0.082	64.47%	199.123	195.800	1.67%
	Ave/Sum	0.189	0.057	69.73%	1914.373	1868.226	2.41%

**Table 16:** Comparison of different thermal comfort models.

Metric	Item	Baseline (Conventional Control)	Probability-Based	ML: SVM-R Only	ML: Hybrid
Comfort level	Mean	0.2217	0.1451	0.0949	0.0943
	SD	(N/A)	(N/A)	0.0053	0.0030
Monthly cooling energy cost (MJ)	Mean	275.970	268.035	270.178	269.987
	SD	(N/A)	(N/A)	4.001	3.086

**Table 17:** SVM-only and hybrid treatment ANOVA test.

Metric (between Groups)	Df	F-Value	*p*-Value	Significant?
Average comfort level	1	5.84	0.0159	Yes
Energy cost	1	0.69	0.4054	No

**Table 18:** Parameters of six subjects picked in main simulation.

No.	Zone	Gender	BMI	Region	Age
1	1	Male	High	United States	Young
2	2	Male	Low		Elder
3	3	Male	Low		Young
4	4	Female	High		Elder
5	5	Female	High		Young
6	6	Female	Low		Elder

**Table 19:** Occupants’ perception and action in College Station, TX.

No.	Mean No. of Manual Actions Taken during Training Period	Thermal Comfort Level in Training	Thermal Comfort Level after Training	Reduction
1	5.9	0.102	0.060	41.6%
2	10.3	0.130	0.086	33.8%
3	8.1	0.120	0.071	40.8%
4	10.1	0.150	0.104	30.7%
5	13.5	0.193	0.084	56.5%
6	20.3	0.258	0.161	37.6%
Average	11.4	0.159	0.094	40.7%

## Data Availability

The data presented in this study are available on request from the corresponding author.
